# Ultrasonic-Assisted Synthesis of N-Doped, Multicolor Carbon Dots toward Fluorescent Inks, Fluorescence Sensors, and Logic Gate Operations

**DOI:** 10.3390/nano12030312

**Published:** 2022-01-18

**Authors:** Jiali Xu, Kai Cui, Tianyu Gong, Jinyang Zhang, Zhirou Zhai, Linrui Hou, Fakhr uz Zaman, Changzhou Yuan

**Affiliations:** School of Materials Science & Engineering, University of Jinan, Jinan 250022, China; Axujl_ME@163.com (J.X.); Acuik_ME@163.com (K.C.); Agongty_ME@163.com (T.G.); Azhangjy_ME@163.com (J.Z.); Azhaizr_ME@163.com (Z.Z.); Azaman_me@163.com (F.u.Z.)

**Keywords:** multicolor carbon dots, kiwifruit, fluorescent inks, fluorescence sensor, logic gate

## Abstract

Over past decades, the multicolor carbon dots (M-CDs) have attracted enormous attentions due to their tunable photoluminescence and versatile applications. Herein, the nitrogen-doped (N-doped) M-CDs including green, chartreuse, and pink emissive CDs are successfully synthesized by ultrasonic treatment of kiwifruit juice with different additive reagents such as ethanol, ethylenediamine, and acetone. Owing to their strong fluorescence upon irradiation with 365 nm UV light, the highly water-soluble M-CDs present great potential in the anticounterfeit field as fluorescent inks. Particularly, the resulting green emission CDs (G-CDs) with excellent fluorescence and stability are applied as a label-free probe model for “on–off” detection of Fe^3+^. The fluorescence of G-CDs is significantly quenched by Fe^3+^ through static quenching. The nanoprobe demonstrates good selectivity and sensitivity toward Fe^3+^ with a detection limit of ~0.11 μM. Besides, the quenched fluorescence of G-CDs by Fe^3+^ can be recovered by the addition of PO_4_^3−^ or ascorbic acid (AA) into the CDs/Fe^3+^ system to realize the “off–on” fluorescent process. Furthermore, NOT and IMPLICATION logic gates are constructed based on the selection of Fe^3+^ and PO_4_^3−^ or AA as the inputs, which makes the G-CD-based sensors utilized as various logic gates at molecular level. Therefore, the N-doped M-CDs hold promising prospects as competitive candidates in monitoring the trace species, applications in food chemistry, anticounterfeit uses, and beyond.

## 1. Introduction

Carbon dots (CDs) have received increasing attention owing to their excellent biocompatibility, good water solubility, low cytotoxicity, robust chemical inertness, high photo/chemical stability, and strong resistance to photobleaching [1,2]. They have especially demonstrated huge potential applications in a wide range of fields, such as sensing, bioimaging, drug delivery, photocatalysis, energy storage, anticounterfeiting, light-emitting diodes (LED), and so on [3,4,5,6,7,8]. Among these applications, great efforts have been devoted to synthesizing the efficient CDs via surface modification and/or heteroatom doping [9,10,11,12,13]. There are two methods for synthesizing CDs, classified as “top-down” and “bottom-up”, respectively. The “top-down” approaches are to cleave the size of large carbon materials, such as laser ablation, chemical oxidation, and arc-discharge, often involving complex reactions [14,15]. Regarding the “bottom-up” methods, the CDs are synthesized via polymerization and carbonization of molecular precursors under specific conditions such as hydrothermal, solvothermal, pyrolysis, microwave, and ultrasonic synthesis, etc. [16,17]. The ultrasound has attracted more attention in recent years due to its high efficiency and the avoidance of using hazardous chemicals and incorporating impurities [18]. The acoustic cavitation effect of the ultrasound is responsible for the formation of CDs in organic reagents [19]. Regretfully, most CDs demonstrate emissions mainly centered at blue or green fluorescence regions [20,21]. The studies on the ultrasonic synthesis of N-doped, multicolor CDs (M-CDs) and even the related physical and chemical performances are still highly desirable.

Compared to the short-wavelength-emissive CDs, the M-CDs have demonstrated numerous fascinating advantages. The adopted avenues to obtain M-CDs are nothing more than adjusting raw materials, reaction conditions (temperatures, reaction times, reaction solvents), and/or separation methods, etc. [22,23,24,25,26,27] C. Dong et al. prepared color-tunable CDs by a one-pot hydrothermal method, and the optimized emission of CDs progressively shifted from green to red with the adjustment of the precursor solution from alkali to acid [22]. S. K. Sahu and coworkers synthesized the multicolor emissive CDs by solvent-controlled and solvent-responded approaches [24]. H. M. Xiong, et al. obtained the full-color fluorescent CDs via silica column chromatography and presented the difference of luminescence resulting from the surface oxidation degree [26]. These pioneering contributions have made significant effects on the development of M-CDs. However, some unavoidable issues, such as the time-consuming process, the cumbersomeness, as well as the fluorescence mechanism of CDs still remained unaddressed. Therefore, it remains a challenging task to develop a facile but efficient strategy to fabricate longer-wavelength-emitting or even M-CDs and further to widen their versatile applications.

Owing to the increasing threat of counterfeiting technologies in medicine, devices, currency, and food products to human life and health, it has triggered the rapid development in nano anticounterfeiting systems [28,29]. Thus far, the security inks have played a significant role in protecting high-value documents, momentous commodities, banknotes, and so on [29]. As a consequence, considerable attention has been paid to the stimuli-responsive fluorescent CDs, which can present a quick response (QR) to external stimuli by emitting different fluorescent colors [30,31]. The distinctive security features of CDs make them more difficult to forge [32,33]. Unfortunately, several technical defects still exist in developing the highly fluorescent CDs.

Simultaneously, the pollutions of metal ions are giving rise to health and environmental concerns [34,35]. Among these ions, Fe^3+^ is an indispensable element for living organisms, while the deficiency or overload of Fe ions in the human body will lead to a risk for diseases, such as heart disorder, liver injury, cancer, etc. [36]. Similarly, some anions and molecules also have immeasurable potential value in the biological, chemical, and environmental systems [37,38]. For instance, the phosphate anion (PO_4_^3^^−^) is one of most momentous constituents of living systems and the related derivatives are of vital importance in biological systems [39], and ascorbic acid (AA), which is well-known as vitamin C and is especially important for the human body [40]. Therefore, the rapid determination of Fe^3+^, PO_4_^3^^−^, as well as AA is highly necessary and desirable in health monitoring [39,40]. In addition, different functional groups on the surfaces of CDs endow them with merits that implement logic gate operations that are responsible for the rapid “turn-on” or “turn-off” fluorescence response to analytes [41,42]. The fluorescence of CDs has especially been recognized as one of the most competitive readout signals for logic gates in recent years [43,44,45]. Based on the change in intensities (absorption or emission) or wavelength (absorption or emission), the logic output can be obtained, and thus, it leads to the successful operation of logic gate via the Boolean arithmetic function [46,47]. However, the tremendous challenge still remains to develop a multiple input CD-based logic gate.

With the comprehensive considerations in mind, in this work, the nitrogen-doped (N-doped) M-CDs including green (G-CDs), chartreuse (C-CDs), and pink (P-CDs) emissive CDs were simply prepared via a facile ultrasonic treatment of kiwifruit juice with different additive reagents changing from ethanol (EA) and ethylenediamine (EDA) to acetone (ACTN). Various characterizations exhibited that the N-doped CDs presented remarkable photostability under ionic strengths, UV light irradiation, and extreme pH. The as-prepared CDs were successfully applied for fluorescent inks, sensors, and logic gates by virtue of their strong and stable photoluminescence (PL) properties, as well as low toxicity.

## 2. Experimental Section

### 2.1. Materials

The kiwifruit was obtained from a local Hualian Supermarket (Jinan, China). NaCl, MgCl_2_·6H_2_O, ZnCl_2_, CuCl_2_·2H_2_O, CaCl_2_, FeCl_3_·6H_2_O, NiCl_2_·6H_2_O, CoCl_2_·6H_2_O, BaCl_2_, CdCl_2_, MnSO_4_·H_2_O, CrCl_3_·6H_2_O, KCl, FeSO_4_·7H_2_O, ethanol (EA), ethylenediamine (EDA), acetone (ACTN), L-Cystein (L-Cys), ascorbic acid (AA), and ammonium phosphate were all purchased from Sinopharm Chemical Reagent Co., Ltd. (Shanghai, China). All chemicals used were of analytical grade and did not require additional purification before use. Ultrasuper water used throughout all experiments was purified by using a Milli-Q Plus system (Millipore).

### 2.2. Preparation of M-CDs

**Scheme 1:**
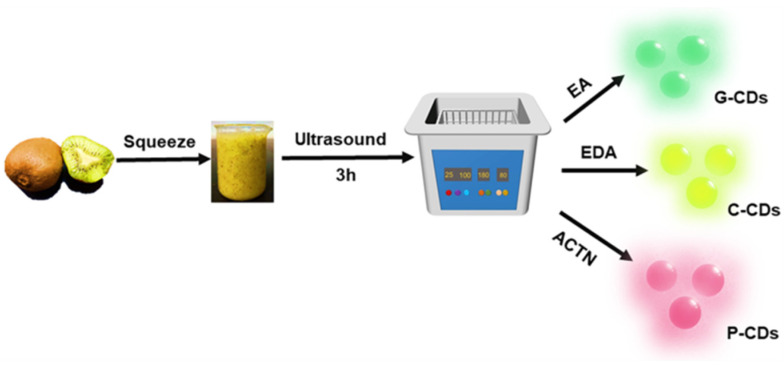
Schematic illustration for facile synthesis of the G-CDs, C-CDs, and P-CDs from kiwi fruit.

M-CDs were synthesized by a simple ultrasonic-assisted treatment of kiwifruit juice with different additives including EA, EDA, and ACTA, as exhibited in Scheme 1. The G-CDs were synthesized by using the following procedures. Typically, the fresh kiwifruit was purchased from a local supermarket, peeled, and then squeezed into a juice through a juicing machine. After filtration, 20 mL of the obtained clear juice and 2 mL of ethanol were mixed well together. Subsequently, the mixture was exposed to high-intensity ultrasonic irradiation (320 W, 80 kHz) at room temperature (RT) in ambient air for 3 h. The resulting solution was centrifuged at 10,000 rpm for 15 min. Afterwards, the supernatant solution was collected, filtered through a filter membrane (0.22 μm), and further purified through a dialysis membrane (1000 MWCO) for 24 h to eliminate the overreacted residues. Finally, the obtained product was dried under vacuum and stored at 4 °C for further use. The C-CDs were prepared with a similar procedure with the G-CDs just by replacing 2 mL of EA with the same volume of EDA. The P-CDs were synthesized in a similar manner with G-CDs through substituting juice (20 mL) and EA (2 mL) with the juice (2 mL) and ACTN (20 mL), respectively.

### 2.3. Fluorescent Ink Evaluation

The as-prepared G-CDs, C-CDs, and P-CDs solutions were further concentrated to obtain fluorescent inks. After cutting a piece from filter paper, the piece was immersed in the G-CDs solution, pulled out, and then dried at RT. Thus, the paper impregnated with G-CDs was gained. Simultaneously, the Chinese characters meaning “Jinan” were written on filter paper with one fountain pen loaded with the solution of C-CDs. Additionally, a picture was drawn on the filter paper by using P-CDs as the pigment. Photos of these filter papers were taken under daylight and UV light (365 nm), respectively, for comparison.

### 2.4. Ion Detection

The detection of ions was performed in aqueous solution at RT with the obtained G-CDs solution (0.5 mg mL^−1^), and the G-CDs were used as a model throughout the entire experiments. Briefly, 1 mL of 1 mM Na^+^, Mg^2+^, Zn^2+^, Cu^2+^, Ca^2+^, Fe^3+^, Ni^2+^, Co^2+^, Ba^2+^, Cd^2+^, Mn^2+^, Cr^3+^, K^+^, Fe^2+^, L-Cys, AA, or PO_4_ ^3−^ was added to the G-CD solution. After a 5 min reaction at RT, the fluorescence spectra were recorded. The selectivity of the G-CDs was explored under the identical conditions. In a typical procedure, 1 mL of the above solutions was mixed separately with the G-CDs solution (3 mL) by gently shaking. All the experiments were carried out under the same conditions, and the corresponding fluorescence spectra were recorded after a 5 min reaction at RT. Furthermore, the sensitivity of the G-CDs for Fe^3+^ was examined via the same procedure by adding aqueous solutions (3 mL) with different concentrations of Fe^3+^. The excited wavelength was set at 360 nm.

### 2.5. Logic Gate Design

For the logic operation, the G-CDs solution (0.5 mg mL^−1^), 1 mM Fe^3+^, 1 mM PO_4_ ^3−^, and 1 mM AA solutions, were used. In the single-input logic operation (NOT), 1 mL of Fe^3+^ was added to the G-CDs solution (3 mL). Similarly, in the dual-input logic operation (IMP), 1 mL of PO_4_ ^3−^ and AA solutions were added to the Fe^3+^-containing G-CDs solution (3 mL), respectively. The quick response (QR) codes were constructed using spreadsheets (https://www.wps.com/) (3 December 2021) [41].

### 2.6. Material Characterization

The size and morphology of the as-synthesized CDs were analyzed using transmission-electron microscopy (TEM, JEOL JEM-2100, Tokyo, Japan). X-ray diffraction (XRD) patterns were recorded on an X-ray diffractometer (Rigaku-TTRIII, Tokyo, Japan). The X-ray photoelectron spectroscopy (XPS) was performed by using an X-ray photoelectron spectrometer (Thermo Escalab 250Xi, Waltham, MA, USA). All fluorescence spectra were performed by a Shimadzu RF-6000 spectrofluorometer equipped with a Xenon arc lamp, and the excitation and emission slits were set at 5 nm (Shimadzu Corporation, Tokyo, Japan). The UV-Vis absorption spectra were recorded with a Shimadzu UV-2600 UV-Vis spectrophotometer (Shimadzu Corporation, Tokyo, Japan). The quantum yield (QY) of CDs was determined with a FS5 fluorescence spectrometer equipped with an SC-30 Integrating Sphere Module (Edinburgh, UK). The fluorescence lifetime was conducted on a DeltaFlex (Horiba Jobin Yvon IBH Ltd., Paris, France).

## 3. Result and Discussion

### 3.1. Physicochemical and Structural Characterizations

Kiwifruit, a fruit with high nutritional value, was used as the precursor for facile fabrication of the CDs. In addition, through a regular adjustment in the added reagents, varying from EA, EDA, and ACTN, the expected M-CDs were successfully prepared. Kiwifruit, as is well known, connately contains carbohydrates, amino acids, vitamin C, and minerals (Ca, K, Se, Zn and Ge) as well as an assortment of phytochemicals (carotenoids), which endows it with an abundance of the elemental C, N, and O. The high nutritional value and unique composition signifies that kiwifruit may be an ideal precursor for developing the N-doped CDs. In this study, G-CDs, C-CDs, and P-CDs with different optical features and surface functional groups were reasonably designed and synthesized.

**Figure 1:**
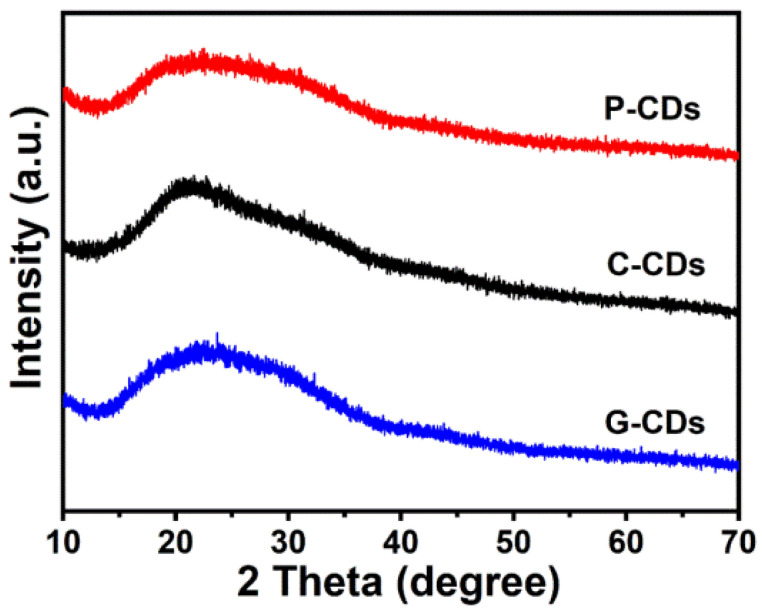
XRD patterns of the G-CDs, C-CDs, and P-CDs.

The X-ray diffraction (XRD) measurement is commonly applied to figure out the structural crystallinity of CDs. Figure 1 shows the XRD patterns of the as-prepared M-CDs. Obviously, all samples demonstrate broad (002) diffraction peaks centered at around 23°, suggesting that the disordered carbon characteristics are probably due to the heteroatom doping [48]. However, the (002) peak shifts to the small angle region by changing the added reagents from EA and EDA to ACTN. This phenomenon may be associated with the doped nitrogen in the carbon core as well as the oxygen-containing functional groups on the surface of the CDs, which is consistent with the findings in the reported literature [25,49].

**Figure 2:**
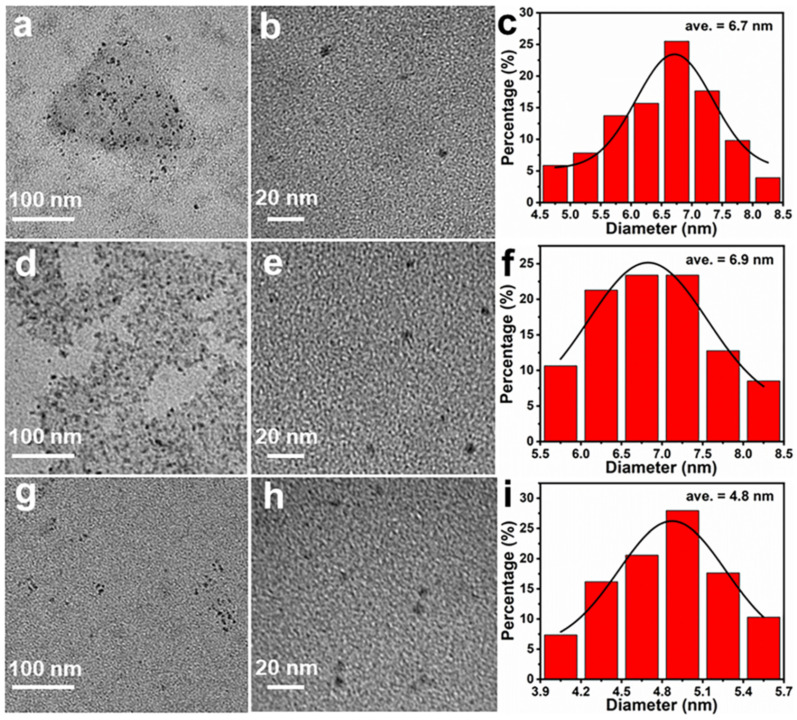
TEM images with different magnifications and corresponding size distribution diagrams of (**a**–**c**) G-CDs, (**d**–**f**) C-CDs, and (**g**–**i**) P-CDs.

The morphologies and particle sizes of M-CDs were obtained by transmission-electron microscopy (TEM), and the corresponding results were collected in Figure 2a–i. The three samples all demonstrate uneven spherical morphologies and all the synthesized dots are uniformly distributed (Figure 2a,b,d,e,g,h). According to the size distribution diagrams, these three samples present average diameters of 6.7 nm (G-CDs) (Figure 2c), 6.9 nm (C-CDs) (Figure 2f) and 4.8 nm (P-CDs) (Figure 2i), indicating the limited influences of the added reagents on the particle sizes of the synthesized samples. Obviously, the sizes of CDs do not increase from G-CDs and C-CDs to P-CDs as the fluorescence emission shifts toward pink, indicating they do not follow the traditional size effect at all. The phenomenon probably arises from the hybridizations of the carbon core and surface states and the conjugation effect of the carbon surface [50,51]. Meanwhile, the formation process of CDs from kiwifruit juice with different additives including EA, EDA, and ACTA was tentatively speculated. Upon ultrasonication, the assembling, crosslinking, and dehydration processes may occur between some organic molecules in kiwifruit juice and the additives to produce some intermediates. Generally, the ultrasonic waves can induce the formation and violent collapse of small vacuum bubbles, from which strong hydrodynamic shear-forces and high-speed impinging liquid jets as well as deagglomeration are produced [19]. Thus, these intermediates can be further carbonized, and N-doped CDs are reasonably prepared due to the presence of the N-containing molecules in kiwifruit juice.

**Figure 3:**
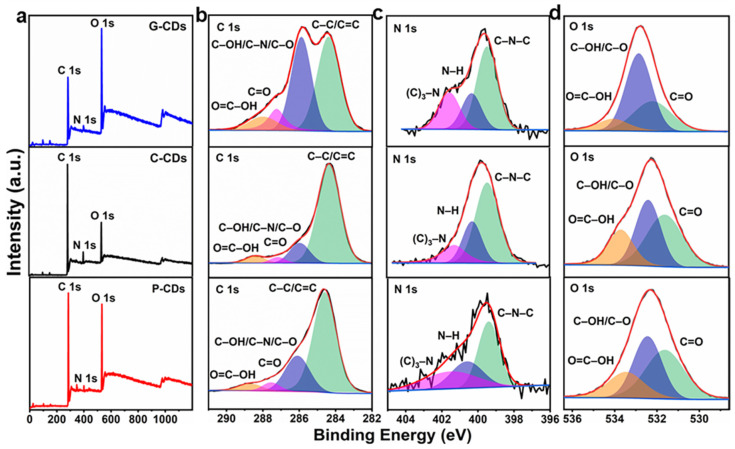
High-resolution (**a**) full survey, (**b**) C 1s, (**c**) N 1s, and (**d**) O 1s XPS spectra of G-CDs (the **upper**), C-CDs (the **middle**), and P-CDs (the **lower**).

X-ray photoelectron spectroscopy (XPS) measurements were performed to characterize the surface state and elemental composition of the as-prepared CDs. The detailed results are displayed in Figure 3. As can be seen from Figure 3a, the survey spectra of the three samples exhibits three typical peaks of C 1s (~285.1 eV), N 1s (~399.6 eV), and O 1s (~532.3 eV), suggesting their same element compositions, i.e., C, O, and N. However, the three-type CDs present different percentages of the atomic contents: C 1s (~64.1%), N 1s (~2.7%), and O 1s (~33.2%) for G-CDs, C 1s (~79.2%), N 1s (~5.7%), and O 1s (~15.1%) for C-CDs, and C 1s (~76.9%), N 1s (~1.7%), and O 1s (~21.0%) for P-CDs (Table S1, ESI). The high-resolution C 1s spectra show the presence of sp^2^ C (C=C), sp^3^ C (C–C/C–N/C–O) and C=O/O=C–OH groups in these CDs (Figure 3b). The deconvoluted five peaks of the C 1 spectra are appropriately located at ~284.6, ~286.1, ~287.5, and ~288.7 eV and are ascribe to the C–C/C=C, C–OH/C–N/C–O, C=O, and O=C–OH groups, respectively [52,53]. Three types of N doping can be affirmed in these CDs by the high-resolution N 1 spectra (Figure 3c). Likely, N 1s can also be divided into three peaks at ~399.4, ~400.2, and ~401.5 eV, corresponding to C–N–C (pyridinic N), N–H (pyrrolic N), and (C)_3_–N (graphitic N), respectively [54]. In addition, the O 1 spectra described in Figure 3d are mainly concentrated at ~531.6, ~532.4, and ~533.5 eV, which are consistent with C=O, C–OH/C–O, and O=C–OH, respectively. The presence of –OH and –COOH groups endows these CDs with excellent water solubility and high selectivity for metal-ion detection [55]. According to the XPS data, the contents of chemical bonds are calculated and summarized (Table S2, ESI). Obviously, all these CDs have the same chemical compositions but different contents of various functional groups, which may be responsible for their unique PL performances [50,51].

### 3.2. Optical Studies

**Figure 4:**
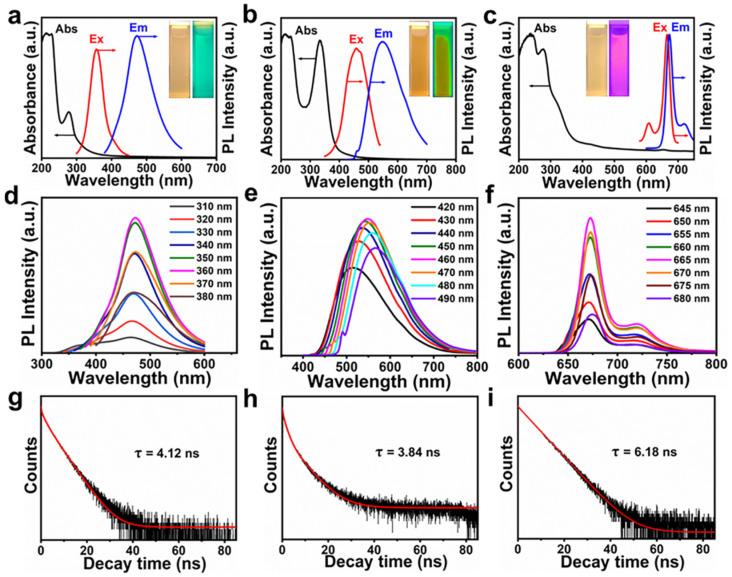
(**a**–**c**) UV-Vis absorption spectra, PL excitation and emission spectra of (**a**) G-CDs, (**b**) C-CDs, and (**c**) P-CDs. The insets are the digital photographs of M-CD aqueous solution under daylight (**left**) and 365 nm UV light (**right**) irradiation. (**d**–**f**) Excitation-dependent PL behaviors and (**g**–**i**) fluorescence lifetime decay curves of (**d**,**g**) G-CDs, (**e**,**h**) C-CDs, and (**f**,**i**) P-CDs.

The optical properties of G-CDs, C-CDs, and P-CDs are further verified by UV-Vis absorption and PL spectra, and the corresponding results are exhibited in Figure 4. As displayed in Figure 4a–c, the UV-Vis absorption spectra of the three CDs display relatively analogous characteristics. Specifically, the peaks at 214 nm (G-CDs), 216 nm (C-CDs), and 238 nm (P-CDs) are ascribed to the π–π* transitions of the aromatic C=C bonds, and the bands at 278 nm (G-CDs), 336 nm (C-CDs), and 275 nm (P-CDs) correspond to the n-π* transition of the conjugated C=O bonds originating from the surface states of the CDs [56]. Likewise, the excitation and emission spectra of the three types of CDs were also optimally conducted (Figure 4a–c). As obtained from the excitation spectra of the three CDs, some differences are apparent. Compared to C-CDs and P-CDs, G-CDs exhibit a relatively narrow excitation spectrum, mainly concentered in the UV region, while those of C-CDs and P-CDs are extended from the UV to the visible region. The maximum excitation/emission peaks of G-CDs are centered at 360/473 nm (Figure 4a), while centered at 460/547 nm for C-CDs (Figure 4b) and 665/673 nm for P-CDs (Figure 4c). Thus, the optimal fluorescence measurements of G-CDs, C-CDs, and P-CDs are examined under the maximum excitation wavelengths of 360, 460, and 665 nm, respectively. The insets exhibit the respective digital photographs of these CD solutions under the irradiation of daylight (left) and 365 nm UV light (right). Apparently, these CD solutions are transparent, light yellow, and clear under the daylight irradiation. These CDs can be well dispersed in water, which is probably attributed to their small particle diameter and abundant surface hydrophilic groups (carbonyl, carboxylic, and hydroxy) [57]. However, a sharp contrast is observed when the UV light of 365 nm is applied to radiate the three aqueous solutions of CDs. The solutions of G-CDs, C-CDs, and P-CDs separately emit green, chartreuse, and pink. The normalized emission spectra of three CDs are displayed in Figure 4a–c. The full width at half maximum (FWHM) values from G-CDs, C-CDs, and P-CDs are 74, 100, and 22 nm, respectively. The narrower the emission peaks of the CDs are, the stronger the color purities become, which is consistent with the prior report [25]. The absolute fluorescence QYs of G-CDs, C-CDs, and P-CDs are simultaneously determined as ~3.7%, ~2.4%, and ~10.0%, respectively.

Furthermore, the PL spectra of the three CDs excited with different wavelengths were also examined (Figure 4d–f). By adjusting the wavelength of the excitation light, G-CDs (Figure 4d) and C-CDs (Figure 4e) both exhibit homologous excitation-dependent fluorescence behaviors, which is similar with the fluorescent CDs reported earlier [57,58]. The excitation wavelengths of the G-CDs (Figure 4a) and C-CDs (Figure 4b) are optimized at 360 nm and 460 nm, respectively. The excitation-dependent fluorescence behavior may result from the optical selection of differently sized nanoparticles or the different energy levels originating from the surface/defects states, which are created by surface groups like C–O, C=O, and –COOH [58]. Interestingly, the fluorescence emission of P-CDs exhibits almost no shift and has only one peak under different excitation wavelengths, revealing the surface state as probably the main reason behind the excitation-independent emission behavior [59,60].

To explore the fluorescence origin of CDs, a universal time-correlated single-photon counting (TCSPC) technique is used to examine the lifetime fluorescence of CDs. The decay curves of the three CDs are shown separately in Figure 4g–i. The fluorescence decay curves of the G-CDs and C-CDs can be well fitted to three kinetic components with a tri-exponential function (Figure 4g,h, Table S3, ESI), suggesting the presence of three different emissive sites in these two CDs. Besides the two short-lived components (*τ*_1_ = 0.21 ns and *τ*_2_ = 1.62 ns for G-CDs, *τ*_1_ = 0.33 ns and *τ*_2_ = 1.65 ns for C-CDs) resulting from the radiative recombination of the excitons, the two decay processes also contain a long-lived component (*τ*_3_ = 4.67 ns for G-CDs, *τ*_3_ = 5.99 ns for C-CDs). The average lifetimes of the G-CDs and C-CDs are calculated to be 4.12 and 3.84 ns, respectively. The multiexponential fitting proves that the multicomponent of CDs, including the graphite center (or conjugated structures) and surface states [58], contribute to the fluorescence emission. The decay curve of P-CDs is well-fitted monoexponentially (Figure 4i). Compared to those of G-CDs and C-CDs, the decay curve of P-CDs only consists of a long-lived component of *τ* = 6.18 ns (Table S1, ESI). The relatively longer fluorescence lifetime of P-CDs indicates that the fluorescence mainly arises from the surface trap recombination [61].

**Figure 5:**
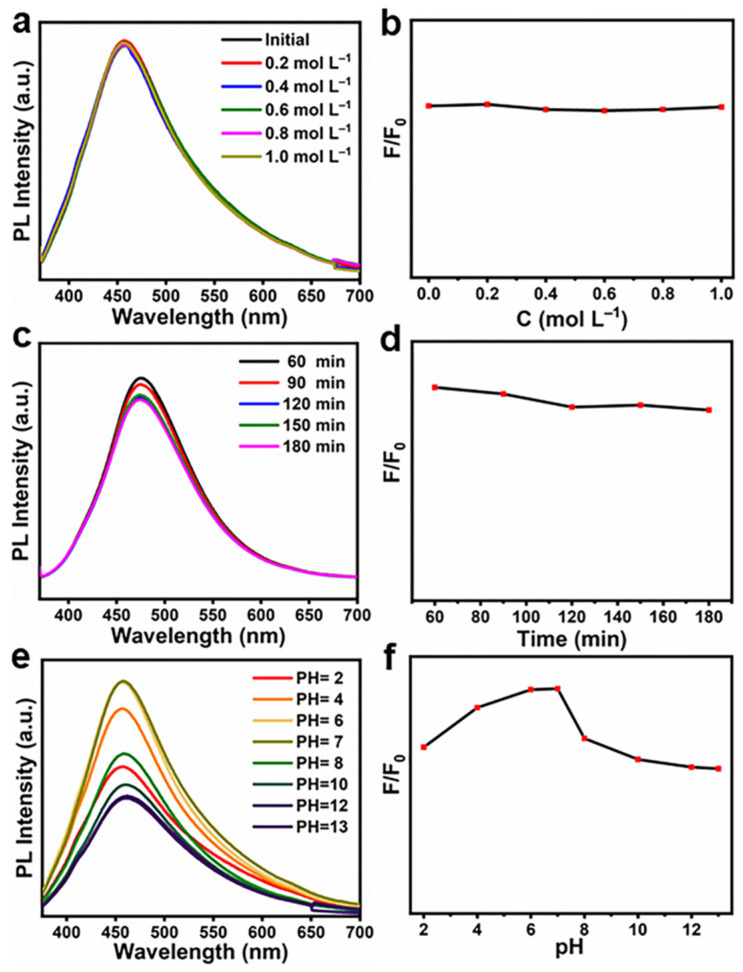
PL spectra and corresponding normalized PL intensities of G-CDs (**a**,**b**) in NaCl solutions with different concentrations, (**c**,**d**) during continuous illumination of UV light, and (**e**,**f**) in aqueous solutions with different pH values ranging from 2 to 13.

Generally, the fluorescence stability is considered as an especially significant factor for their potential applications. Hence, the PL performances of the CD solution encountering different conditions are examined in detail by using the G-CDs as a model, and the related measurement results are displayed in Figure 5. The influences of ionic strength and UV exposure on the stability of the PL are firstly investigated (Figure 5a–d). Obviously, there are no significant changes in the peak characteristics (Figure 5a,c). As can be seen from the PL data (Figure 5a,b), the PL intensity shows almost no obvious differences with the increase in the surrounding ionic strengths by regulating the concentrations of NaCl from 0 to 1 mol L^−1^. Likewise, no serious PL bleaching is observed after continuous UV-light irradiation for 180 min (Figure 5c,d). As for the effects of pH, the fluorescence response of CDs becomes even stronger with the increase in the surrounding pH values in the acidic environment, but shows little change in a neutral or alkaline environment (Figure 5e,f). These results highlight that these CDs demonstrate excellent light stability and photobleaching resistance, which are beneficial for their potential applications in fluorescent labels, fluorescent inks, and other fields.

### 3.3. Fluorescent Ink Properties

**Figure 6:**
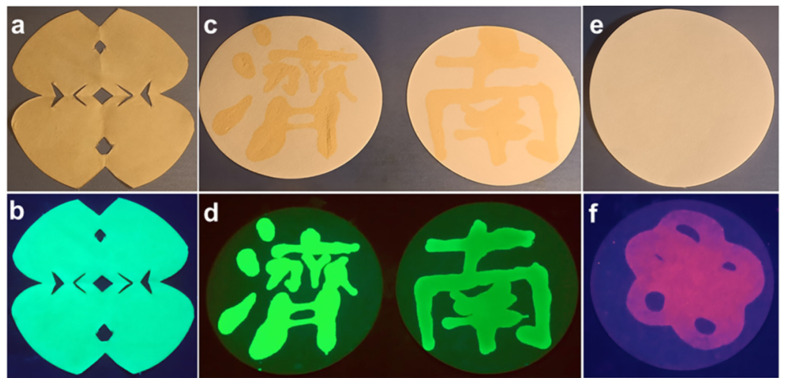
The photographic illustration images of (**a**,**b**) G-CDs, (**c**,**d**) C-CDs, and (**e**,**f**) P-CDs under (**a**,**c**,**e**) the daylight and (**b**,**d**,**f**) 365 nm UV-light on filter paper, respectively.

In view of the unique fluorescence properties including good dispersibility and strong fluorescence and stability, the as-prepared M-CDs are further used as invisible fluorescent inks. Three representation patterns of cutting, writing, and drawing with filter paper are adopted to make them feasible candidates for fluorescent ink applications, and the corresponding results are shown in Figure 6a–f. As displayed in Figure 6a,c,e, there are no visible patterns appearing on the filter paper under the irradiation of daylight, although these filter papers are treated with the as-prepared M-CDs. More interestingly, when excited by the UV light of 365 nm, the filter paper coated with the G-CD solution exhibited green emission (Figure 6b). Visually, the chartreuse Chinese characters of jinan (“济南”) appear in the writing marks with C-CDs as inks (Figure 6d). Simultaneously, a clear, bright pink-emitting picture also emerges on the filter paper drawn with P-CDs as a pigment (Figure 6f). Therefore, these M-CDs can be widely used as various anticounterfeit marks, which have a promising application in the security field.

### 3.4. Sensing Applications

**Figure 7:**
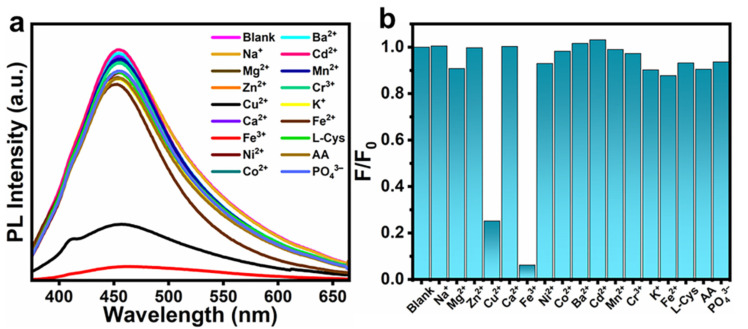
(**a**) PL spectra of the G-CDs aqueous solutions in the presence of different ions and molecules. (**b**) *F*/*F*_0_ of the G-CD solution with different ions and molecules. *F*_0_ and *F* for the PL intensities in the absence and presence of different ions and molecules, respectively.

In general, the surface of the biomass-derived CDs reserve the oxygen-containing functional groups, including hydroxyl, carboxyl, and amino groups, and these functional groups can coordinate with the detection object to cause corresponding changes in fluorescence [52,62]. In view of these characteristics, the CDs can serve as a potential sensing candidate, implying their future application in the sensor field. With regards to this, the G-CDs are applied as a model to investigate the fluorescence responses to the interferents including Na^+^, Mg^2+^, Zn^2+^, Cu^2+^, Ca^2+^, Fe^3+^, Ni^2+^, Co^2+^, Ba^2+^, Cd^2+^, Mn^2+^, Cr^3+^, K^+^, Fe^2+^, L-Cys, AA, and PO_4_^3−^ under the same conditions (Figure 7). Accordingly, the PL spectra of CDs after the addition of 1 mM interferents as mentioned above (Figure 7a), the obtained F/F_0_ bar diagram in the absence and presence of different interferents are shown in Figure 7b. Obviously, Fe^3+^ has the greatest fluorescence-quenching effect among the ions, suggesting that the CDs are more sensitive and reliable for the detection of Fe^3+^ ion than other ions.

**Figure 8:**
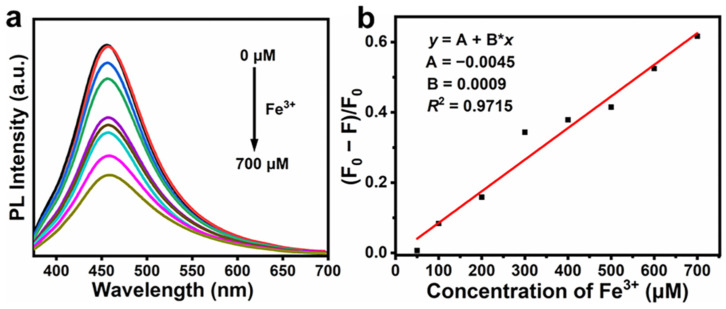
(**a**) PL spectra and (**b**) quenching efficiency of the G-CDs with different concentrations of Fe^3+^.

Excellent fluorescent probes should not only have specific selectivity but also possess high sensitivity. Figure 8a shows the changes in the fluorescence spectra of the G-CD aqueous solution in the presence of different concentrations of Fe^3+^ (0, 50, 100, 200, 300, 400, 500, 600, and 700 μM). It clearly demonstrates that the fluorescence can be quenched gradually along with the increase in the concentration of Fe^3+^, suggesting the feasibility of Fe^3+^ detection. The quenching efficiency, i.e., (*F*_0_ − *F*)/*F*_0_, especially exhibits a good linearity with the concentrations of Fe^3+^ within the concentration range of 50 to 700 μM (*R*^2^ = 0.9715) (Figure 8b). Here, *F*_0_ and *F* present the highest PL intensity, excited at 360 nm in the absence and presence of Fe^3+^, respectively. Iron is an especially important element for life; however, once Fe^3+^ is out of the effective concentration range it causes diseases. The lowest limit of detection (LOD) of the Fe^3+^ is also extremely significant for Fe^3+^ sensor. The LOD of G-CDs toward Fe^3+^ is estimated to be 0.11 μM, which is calculated according to the equation LOD = 3*δ*/*S* (a signal-to-noise ratio of S/N = 3), where *δ* and *S* are the standard deviation and the slope of linear range, respectively [63]. Overall, the G-CD fluorescence-sensing probe manifests excellent performances including the wide-detection range, and high selectivity/sensitivity toward Fe^3+^ in the aqueous solution. These results reveal that G-CDs can be applied as an efficient fluorescence sensor for trace amounts of Fe^3+^ detection in biomedical and environmental systems.

### 3.5. Quenching Mechanism

**Figure 9:**
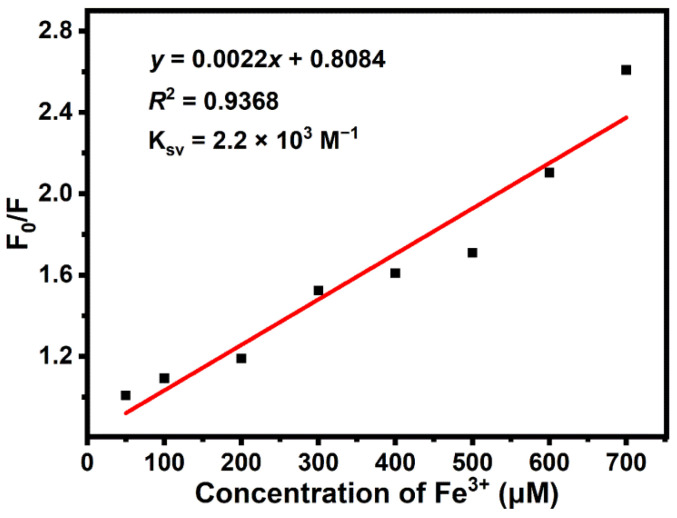
Stern–Volmer plot between *F*_0_/*F* and [*Q*].

The fluorescence-quenching process involves the interaction between the quencher molecules and fluorescent molecules. Normally, two quenching forms including dynamic and static quenching mechanisms are widely acknowledged, and the type of quenching mechanism can be determined by the quenching constant from the standard Stern–Volmer equation, i.e., *F*_0_/*F* = 1 + *K_sv_*[*Q*], where *F*_0_ and *F* represent the fluorescence intensities in the absence and presence of quenchers, respectively, [*Q*] is the concentration of the quencher, and *K_sv_* is the quenching constant [64]. The dynamic quenching is mainly related to diffusion, and the *K_sv_* of the fluorescent materials may increase along with the rise in temperature, where the maximum scatter-collision-quenching constant by all kinds of quenchers is 2.0 × 10^10^ L mol^−1^ s^−1^ [65]. As for the static quenching mechanism, the *K_sv_* values follow the descending trend with the increase in the temperature. To pursue the quenching mechanism of G-CDs, the quenching constant is calculated by the standard Stern–Volmer equation. As shown in Figure 9, the Stern–Volmer plot (*F*_0_/*F*) exhibits a good linear relationship with the concentration of Fe^3+^. The *K_sv_* is calculated as 2.2 × 10^3^ M^−1^, which is larger than the limited dynamic-quenching constant (2.0 × 10^3^ M^−1^). Thus, a static-quenching mechanism is reasonably recognized in the system, revealing the formation of nonfluorescent ground-state complexes between CDs and the quencher. The Fe^3+^ can chelate or coordinate with the oxygenated and nitrogenous functional groups on the surface of the G-CDs to form the complexes. Thus, it changes the distribution of the energy states and enhances the nonradiative recombination of electron/hole, subsequently resulting in the PL quenching [62,63,64,65,66].

**Figure 10:**
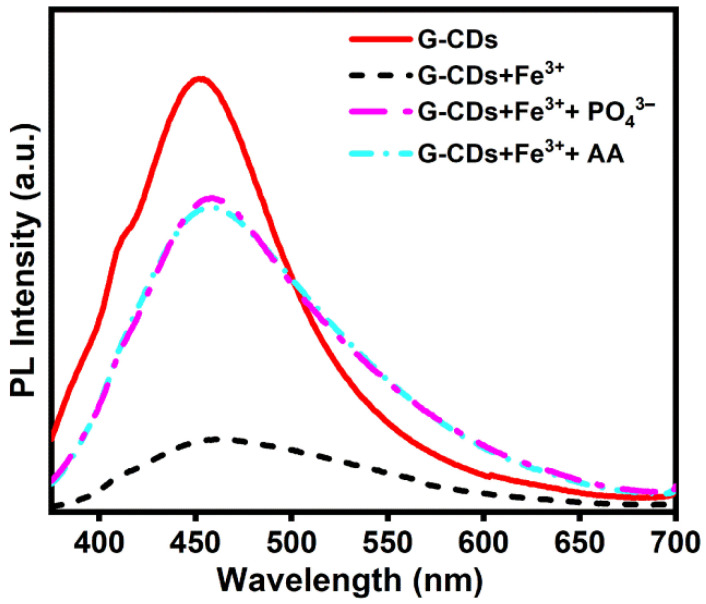
PL-intensity spectra of fluorescence quenching and recovery.

**Figure 11:**
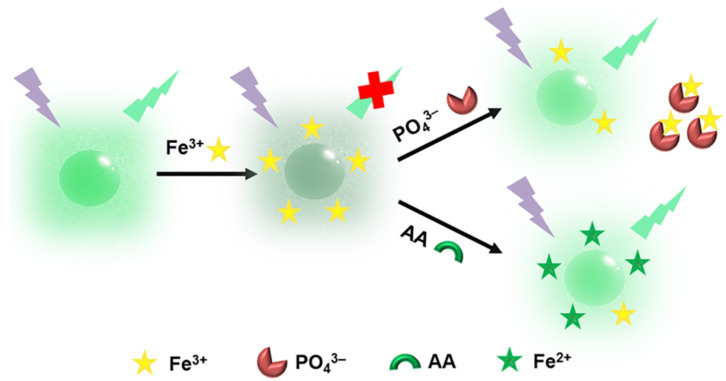
Schematic illustration for the “off–on” fluorescence-sensing mechanisms towards Fe^3+^, PO_4_^3−^, and AA based on G-CDs.

In addition to the sensing of metal ions, the capable sensing of the G-CDs/Fe^3+^ system toward anions or molecules was also designed based on the “on–off” and “off–on” characters of the fluorescence (Figure 10). As displayed in Figure 10, the PL intensity of the G-CDs shows an obvious decrease with the addition of Fe^3+^, which presents the typical “on–off” fluorescence performance (Figure 11). The fluorescence quenching may originate from the formation of a nonfluorescent complex between the surface oxygen- (or nitrogen-) containing groups in the CDs and Fe^3+^ [67], which further confirms the static fluorescence mechanism of CDs (Figure 11). Notably, a significant PL recovery can be achieved with the subsequent addition of PO_4_^3−^ (1 mM) into the G-CD solution containing 700 µM Fe^3+^, thus exhibiting an “off–on” fluorescence character. The stripping of Fe^3+^ by the added PO_4_^3−^ from the surface of CDs is probably responsible for such fluorescence recovery, and the related off–on fluorescence process is schematically shown in Figure 11. Additionally, the molecule of AA also has the same fluorescence-recovery function as that of the PO_4_^3−^ (Figure 10). Namely, the quenched fluorescence of CDs by Fe^3+^ can be recovered by the addition of AA. This phenomenon is rationally ascribed to the transformation from Fe^3+^ to Fe^2+^, resulting from the efficient reduction of AA (Figure 11), while the CDs show much higher selectivity toward Fe^3+^ than Fe^2+^ [68]. The results here feature that the G-CDs can be utilized towards the detection of Fe^3+^, PO_4_^3−^, and AA.

### 3.6. Logic Gate Operation

**Figure 12:**
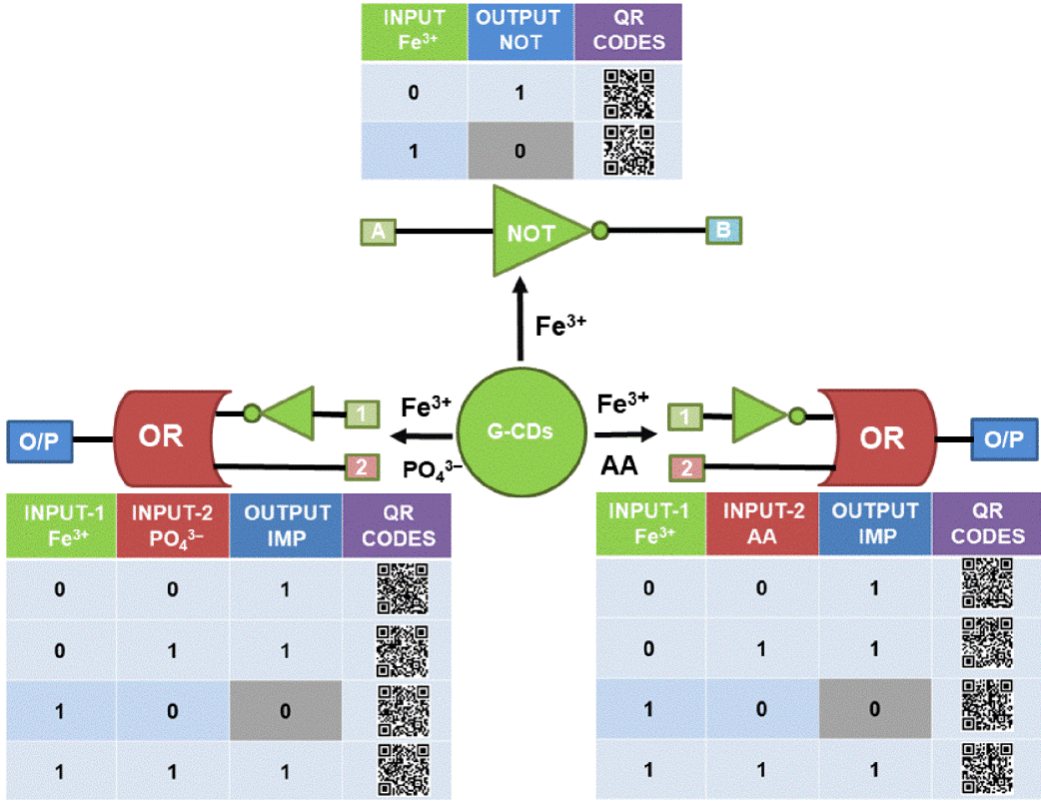
A NOT logic gate and two IMPLICATION logic gates and their truth tables.

In view of the sensitive response of the as-prepared CDs towards Fe^3+^, PO_4_^3−^, and AA, a fluorometric logic system with a function of executing a single-input logic operation (NOT) and dual-input logic operation (IMP) is reasonably designed by using the G-CDs as a model (Figure 12). In this devised logic system, the G-CDs bear the responsibility of logic gates, while the ions (Fe^3+^ and PO_4_^3−^) and molecule (AA) are applied as the chemical inputs, and the fluorescence signals are regarded as the output. To perform the logic gates even better, the absence and presence of these inputs are marked as Boolean logic functions of “0” and “1” states, respectively. Simultaneously, the outputs defined as “0” and “1” separately correspond to the quenched fluorescence and the maximum fluorescence. The corresponding parameters and results of each logic operation are encoded in QR codes with unique spreadsheets, and the related QR codes are wholly exhibited in truth tables (Movie S1, ESI). As for the single-input logic operation, the G-CDs serve as the gate, while Fe^3+^ is used as the chemical input. The addition of Fe^3+^ toward the G-CD system (input = 1) results in their obvious quenching of fluorescence (output = 0), which is in accord with the single-input-driven logic operation of NOT.

Further, the IMP logic gate with Fe^3+^ and PO_4_^3−^ as inputs 1 and 2, respectively, to G-CDs is constructed. The absence of both inputs (input = 0, 0) and even the individual input of PO_4_^3−^ (input = 0, 1) cannot cause the significant change of fluorescence (output = 1). However, the fluorescence intensity is substantially quenched (output = 0) only in the presence of Fe^3+^ (input = 1, 0). Interestingly, the continuous addition of PO_4_^3−^ (input = 1, 1) can trigger a remarkable recovery of fluorescence (output = 1). Similarly, the multiple input logic gate IMP is also achieved by using Fe^3+^ and AA as inputs 1 and 2, respectively. Obviously, only in the presence of Fe^3+^ and absence of AA (input = 1, 0), the prominent fluorescence quenching is observed (output = 0), while in the case of other inputs (0/0, 0/1, 1/1), the CD system still demonstrates strong fluorescence performance (output = 1). The responses agree well with the dual-input-driven logic operation, IMP.

## 4. Conclusions

In conclusion, in the work, we developed a facile yet effective ultrasonic-assisted synthesis methodology to directly fabricate small-size and strong fluorescent N-doped M-CDs from the nutritious and naturally renewable kiwifruit. The emission colors of M-CDs changed from green and chartreuse to pink just through a regular adjustment in the added reagents varying from ethanol and ethylenediamine to acetone. The as-prepared N-doped CDs exhibited excellent chemical and optical stabilities. It is these appealing properties and superiorities that make the resulting M-CDs smart platforms for fluorescent inks, as well as sequential fluorescent probes for the determination of Fe^3+^ and PO_4_^3−^/or AA. Furthermore, the fluorescent nanoprobers were successfully utilized for the construction of molecular logic gates and for imaging Fe^3+^ and PO_4_^3−^/AA. More significantly, we strongly believe that the ultrasonic-assisted synthesis concept here can be extended to other biomasses for advanced heteroatom-doped CDs towards fluorescent inks/sensors, logic gates, and beyond.

## Data Availability

Not applicable.

## Supplementary Materials

The following supporting information can be downloaded at: https://www.mdpi.com/article/10.3390/nano12030312/s1, Table S1: The atomic contents from the XPS data; Table S2: XPS data analysis of the C 1s spectra of three samples; Table S3: Fitting parameters and average PL lifetimes (τ) of the G-CDs, C-CDs, and P-CDs; Video S1: Movie S1.

## References

[1] Yu J., Yong X., Tang Z., Yang B., Lu S. (2021). Theoretical understanding of structure–property relationships in luminescence of carbon dots. J. Phys. Chem. Lett..

[2] Javed N., O’Carroll D.M. (2021). Carbon dots and stability of their optical properties. Part. Part. Syst. Charact..

[3] Liu J., Li R., Yang B. (2020). Carbon dots: A new type of carbon-based nanomaterial with wide applications. ACS Cent. Sci..

[4] Guo R., Li L., Wang B., Xiang Y., Zou G., Zhu Y., Hou H., Ji X. (2021). Functionalized carbon dots for advanced batteries. Energy Storage Mater..

[5] Li X.C., Zhao S.J., Li B.L., Yang K., Lan M.H., Zeng L.T. (2021). Advances and perspectives in carbon dot-based fluorescent probes: Mechanism, and application. Coord. Chem. Rev..

[6] Tian L., Li Z., Wang P., Zhai X., Wang X., Li T. (2021). Carbon quantum dots for advanced electrocatalysis. J. Energy Chem..

[7] Wang B., Song H., Qu X., Chang J., Yang B., Lu S. (2021). Carbon dots as a new class of nanomedicines: Opportunities and challenges. Coord. Chem. Rev..

[8] Kumar V.B., Sahu A.K., Mohsin A.S.M., Li X., Gedanken A. (2017). Refractive-index tuning of highly fluorescent carbon dots. ACS Appl. Mater. Interfaces.

[9] Bourlinos A.B., Bakandritsos A., Kouloumpis A., Gournis D., Krysmann M., Giannelis E.P., Polakova K., Safarova K., Hola K., Zboril R. (2012). Gd(III)-doped carbon dots as a dual fluorescent-MRI probe. J. Mater. Chem..

[10] Sun D., Ban R., Zhang P.-H., Wu G.-H., Zhang J.-R., Zhu J.-J. (2013). Hair fiber as a precursor for synthesizing of sulfur- and nitrogen-co-doped carbon dots with tunable luminescence properties. Carbon.

[11] Hou X., Hu Y., Wang P., Yang L., Al Awak M.M., Tang Y., Twara F.K., Qian H., Sun Y.-P. (2017). Modified facile synthesis for quantitatively fluorescent carbon dots. Carbon.

[12] Kumar V.B., Sheinberger J., Porat Z., Shav-Tal Y., Gedanken A. (2016). A hydrothermal reaction of an aqueous solution of BSA yields highly fluorescent N doped C-dots used for imaging of live mammalian cells. J. Mater. Chem. B.

[13] Nissan I., Kumar V.B., Porat Z.E., Makovec D., Shefi O., Gedanken A. (2017). Sonochemically-fabricated Ga@C-dots@Ga nanoparticle-aided neural growth. J. Mater. Chem. B.

[14] Qi C., Wang H., Yang A., Wang X., Xu J. (2021). Facile fabrication of highly fluorescent N-Doped carbon quantum dots using an ultrasonic-assisted hydrothermal method: Optical properties and cell Imaging. ACS Omega.

[15] Zhang Y., Park M., Kim H.Y., Ding B., Park S.-J. (2017). A facile ultrasonic-assisted fabrication of nitrogen-doped carbon dots/BiOBr up-conversion nanocomposites for visible light photocatalytic enhancements. Sci. Rep..

[16] Ma Z., Ming H., Huang H., Liu Y., Kang Z. (2012). One-step ultrasonic synthesis of fluorescent N-doped carbon dots from glucose and their visible-light sensitive photocatalytic ability. New J. Chem..

[17] Kumar V.B., Perelshtein I., Lipovsky A., Porat Z.e., Gedanken A. (2015). The sonochemical synthesis of Ga@C-dots particles. RSC Adv..

[18] Kumar V.B., Kumar R., Gedanken A., Shefi O. (2019). Fluorescent metal-doped carbon dots for neuronal manipulations. Ultrason. Sonochem..

[19] Kumar V.B., Porat Z.e., Gedanken A. (2016). Facile one-step sonochemical synthesis of ultrafine and stable fluorescent C-dots. Ultrason. Sonochem..

[20] Liu S., Tian J., Wang L., Luo Y., Zhai J., Sun X. (2011). Preparation of photoluminescent carbon nitride dots from CCl_4_ and 1,2-ethylenediamine: A heat-treatment-based strategy. J. Mater. Chem..

[21] Deng X., Wu D. (2014). Highly sensitive photoluminescence energy transfer detection for 2,4,6-trinitrophenol using photoluminescent carbon nanodots. RSC Adv..

[22] Jiao Y., Liu Y., Meng Y., Gao Y., Lu W., Liu Y., Gong X., Shuang S., Dong C. (2020). Novel processing for color-tunable luminescence carbon dots and their advantages in biological systems. ACS Sustain. Chem. Eng..

[23] Huo F., Liu Y., Zhu M., Gao E., Zhao B., Yang X. (2019). Ultrabright full color carbon dots by fine-tuning crystal morphology controllable synthesis for multicolor bioimaging and sensing. ACS Appl. Mater. Interfaces.

[24] Kumari R., Sahu S.K. (2020). Effect of solvent-derived highly luminescent multicolor carbon dots for white-light-emitting diodes and water detection. Langmuir.

[25] Zheng Y., Arkin K., Hao J., Zhang S., Guan W., Wang L., Guo Y., Shang Q. (2021). Multicolor carbon dots prepared by single-factor control of graphitization and surface oxidation for high-quality white light-emitting diodes. Adv. Opt. Mater..

[26] Ding H., Yu S.-B., Wei J.-S., Xiong H.-M. (2016). Full-color light-emitting carbon dots with a surface-state-controlled luminescence mechanism. ACS Nano.

[27] Huo X., Shen H., Liu R., Shao J. (2021). Solvent effects on fluorescence properties of carbon dots: Implications for multicolor imaging. ACS Omega.

[28] Bae H.J., Bae S., Park C., Han S., Kim J., Kim L.N., Kim K., Song S.-H., Park W., Kwon S. (2015). Biomimetic microfingerprints for anti-counterfeiting strategies. Adv. Mater..

[29] Hou X., Ke C., Bruns C.J., McGonigal P.R., Pettman R.B., Stoddart J.F. (2015). Tunable solid-state fluorescent materials for supramolecular encryption. Nat. Commun..

[30] Cai Y., Huang Z., Cheung M.H.C., Motto-Ros V., Chu P.-C., Wang Y., Zhong H., Yuen R., Leung K.S.Y., Lum J.T.S. (2016). Elemental analysis of Chinese black inks on xuan paper by ArF laser-excited plume fluorescence. Anal. Chem..

[31] Chen Z., Zhao Z., Wang Z., Zhang Y., Sun X., Hou L., Yuan C. (2018). Foxtail millet-derived highly fluorescent multi-heteroatom doped carbon quantum dots towards fluorescent inks and smart nanosensors for selective ion detection. New J. Chem..

[32] Khan W.U., Wang D., Zhang W., Tang Z., Ma X., Ding X., Du S., Wang Y. (2017). High quantum yield green-emitting carbon dots for Fe(III) detection, biocompatible fluorescent ink and cellular imaging. Sci. Rep..

[33] Wang B., Song A., Feng L., Ruan H., Li H., Dong S., Hao J. (2015). Tunable amphiphilicity and multifunctional applications of ionic-liquid-modified carbon quantum dots. ACS Appl. Mater. Interfaces.

[34] Zhang H.-Y., Wang Y., Xiao S., Wang H., Wang J.-H., Feng L. (2017). Rapid detection of Cr(VI) ions based on cobalt(II)-doped carbon dots. Biosens. Bioelectron..

[35] Bagheri Z., Ehtesabi H., Rahmandoust M., Ahadian M.M., Hallaji Z., Eskandari F., Jokar E. (2017). New insight into the concept of carbonization degree in synthesis of carbon dots to achieve facile smartphone based sensing platform. Sci. Rep..

[36] Cui X., Wang Y., Liu J., Yang Q., Zhang B., Gao Y., Wang Y., Lu G. (2017). Dual functional N- and S-co-doped carbon dots as the sensor for temperature and Fe^3+^ ions. Sens. Actuators B Chem..

[37] Yang X.-F., Wang L., Xu H., Zhao M. (2009). A fluorescein-based fluorogenic and chromogenic chemodosimeter for the sensitive detection of sulfide anion in aqueous solution. Anal. Chim. Acta.

[38] Simões E.F.C., da Silva L.P., da Silva J.C.G.E., Leitão J.M.M. (2020). Hypochlorite fluorescence sensing by phenylboronic acid-alizarin adduct based carbon dots. Talanta.

[39] Liu Y., Liu Y., Park S.-J., Zhang Y., Kim T., Chae S., Park M., Kim H.-Y. (2015). One-step synthesis of robust nitrogen-doped carbon dots: Acid-evoked fluorescence enhancement and their application in Fe^3+^ detection. J. Mater. Chem. A.

[40] Du F., Gong X., Lu W., Liu Y., Gao Y., Shuang S., Xian M., Dong C. (2018). Bright-green-emissive nitrogen-doped carbon dots as a nanoprobe for bifunctional sensing, its logic gate operation and cellular imaging. Talanta.

[41] Xiao M., Zhou Q., Zhang H., Zhou L., Ma J., Yi C. (2021). Logic gate design using multicolor fluorescent carbon nanodots for smartphone-based information extraction. ACS Appl. Nano Mater..

[42] Dhenadhayalan N., Lin K.-C. (2015). Chemically induced fluorescence switching of carbon-dots and its multiple logic gate implementation. Sci. Rep..

[43] Zou W.-S., Zhao Q.-C., Kong W.-L., Wang X.-F., Chen X.-M., Zhang J., Wang Y.-Q. (2018). Multi-level fluorescent logic gate based on polyamine coated carbon dots capable of responding to four stimuli. Chem. Eng. J..

[44] Yuan Y., Zhao X., Liu S., Li Y., Shi Y., Yan J., Hu X. (2016). A fluorescence switch sensor used for D-Penicillamine sensing and logic gate based on the fluorescence recovery of carbon dots. Sens. Actuators B Chem..

[45] Liu Y., Zhan G., Liu Z.-W., Bian Z.-Q., Huang C.-H. (2016). Room-temperature phosphorescence from purely organic materials. Chin. Chem. Lett..

[46] Gao Y., Jiao Y., Lu W., Liu Y., Han H., Gong X., Xian M., Shuang S., Dong C. (2018). Carbon dots with red emission as a fluorescent and colorimeteric dual-readout probe for the detection of chromium(VI) and cysteine and its logic gate operation. J. Mater. Chem. B.

[47] Zhang L., Qin J., Yang Q., Wei S., Yang R. (2019). Cost-effective and facile fluorescent probes for label-free recognition of chlorpromazine hydrochloride and logic gate operation. J. Photochem. Photobiol. A.

[48] Li M., Hu C., Yu C., Wang S., Zhang P., Qiu J. (2015). Organic amine-grafted carbon quantum dots with tailored surface and enhanced photoluminescence properties. Carbon.

[49] Wang Z., Shen J., Xu B., Jiang Q., Ming S., Yan L., Gao Z., Wang X., Zhu C., Meng X. (2021). Thermally driven amorphous-crystalline phase transition of carbonized polymer dots for multicolor room-temperature phosphorescence. Adv. Opt. Mater..

[50] Ai L., Yang Y., Wang B., Chang J., Tang Z., Yang B., Lu S. (2021). Insights into photoluminescence mechanisms of carbon dots: Advances and perspectives. Sci. Bull..

[51] Gharat P.M., Chethodil J.M., Srivastava A.P., Praseetha P.K., Pal H., Choudhury S.D. (2019). An insight into the molecular and surface state photoluminescence of carbon dots revealed through solvent-induced modulations in their excitation wavelength dependent emission properties. Photochem. Photobiol. Sci..

[52] Jia J., Lin B., Gao Y., Jiao Y., Li L., Dong C., Shuang S. (2019). Highly luminescent N-doped carbon dots from black soya beans for free radical scavenging, Fe^3+^ sensing and cellular imaging. Spectrochim. Acta Part A.

[53] Atchudan R., Edison T.N., Perumal S., Vinodh R., Sundramoorthy A.K., Babu R.S., Lee Y.R. (2021). Leftover kiwi fruit peel-derived carbon dots as a highly selective fluorescent sensor for detection of ferric ion. Chemosensors.

[54] Ding H., Ji Y., Wei J.-S., Gao Q.-Y., Zhou Z.-Y., Xiong H.-M. (2017). Facile synthesis of red-emitting carbon dots from pulp-free lemon juice for bioimaging. J. Mater. Chem. B.

[55] Wang C., Shi H., Yang M., Yan Y., Liu E., Ji Z., Fan J. (2020). Facile synthesis of novel carbon quantum dots from biomass waste for highly sensitive detection of iron ions. Mater. Res. Bull..

[56] Huang Q., Li Q., Chen Y., Tong L., Lin X., Zhu J., Tong Q. (2018). High quantum yield nitrogen-doped carbon dots: Green synthesis and application as “off-on” fluorescent sensors for the determination of Fe^3+^ and adenosine triphosphate in biological samples. Sens. Actuators B Chem..

[57] Zhao D., Liu X., Wei C., Qu Y., Xiao X., Cheng H. (2019). One-step synthesis of red-emitting carbon dots via a solvothermal method and its application in the detection of methylene blue. RSC Adv..

[58] Khan Z.M.S.H., Rahman R.S., Shumaila, Islam S., Zulfequar M. (2019). Hydrothermal treatment of red lentils for the synthesis of fluorescent carbon quantum dots and its application for sensing Fe^3+^. Opt. Mater..

[59] Singh B., Bahadur R., Rangara M., Gandhi M.N., Srivastava R. (2021). Influence of surface states on the optical and cellular property of thermally stable red emissive graphitic carbon dots. ACS Appl. Bio Mater..

[60] Liu Y., Zhou L., Li Y., Deng R., Zhang H. (2017). Highly fluorescent nitrogen-doped carbon dots with excellent thermal and photo stability applied as invisible ink for loading important information and anti-counterfeiting. Nanoscale.

[61] Yuan F., Wang Y.-K., Sharma G., Dong Y., Zheng X., Li P., Johnston A., Bappi G., Fan J.Z., Kung H. (2020). Bright high-colour-purity deep-blue carbon dot light-emitting diodes via efficient edge amination. Nat. Photonics.

[62] Liu Y., Liu Y., Park M., Park S.-J., Zhang Y., Akanda M.R., Park B.-Y., Kim H.Y. (2017). Green synthesis of fluorescent carbon dots from carrot juice for in vitro cellular imaging. Carbon Lett..

[63] Picard M., Thakur S., Misra M., Mohanty A.K. (2019). Miscanthus grass-derived carbon dots to selectively detect Fe^3+^ ions. RSC Adv..

[64] Shangguan J., Huang J., He D., He X., Wang K., Ye R., Yang X., Qing T., Tang J. (2017). Highly Fe^3+^-selective fluorescent nanoprobe based on ultrabright N/P codoped carbon dots and its application in biological samples. Anal. Chem..

[65] Qi H., Teng M., Liu M., Liu S., Li J., Yu H., Teng C., Huang Z., Liu H., Shao Q. (2019). Biomass-derived nitrogen-doped carbon quantum dots: Highly selective fluorescent probe for detecting Fe^3+^ ions and tetracyclines. J. Colloid Interface Sci..

[66] Cheng C., Xing M., Wu Q. (2019). A universal facile synthesis of nitrogen and sulfur co-doped carbon dots from cellulose-based biowaste for fluorescent detection of Fe^3+^ ions and intracellular bioimaging. Mater. Sci. Eng. C.

[67] Amin N., Afkhami A., Hosseinzadeh L., Madrakian T. (2018). Green and cost-effective synthesis of carbon dots from date kernel and their application as a novel switchable fluorescence probe for sensitive assay of Zoledronic acid drug in human serum and cellular imaging. Anal. Chim. Acta.

[68] Guo Z., Liu X., Yu H., Hou F., Gao S., Zhong L., Xu H., Yu Y., Meng J., Wang R. (2021). Continuous response fluorescence sensor for three small molecules based on nitrogen-doped carbon quantum dots from prunus lannesiana and their logic gate operation. Spectrochim. Acta Part A.

