# Supplementary material for: Ultrasonic-Assisted Synthesis of N-Doped, Multicolor Carbon Dots toward Fluorescent Inks, Fluorescence Sensors, and Logic Gate Operations
Source: Nanomaterials (Basel). 2022 Jan 18;12(3):312. doi: 10.3390/nano12030312 (PMC8839126; doi:10.3390/nano12030312)
Supplement: Supplementary file 1 [file nanomaterials-12-00312-s001.zip › SI_nanomaterials-1529809.pdf]

nanomaterials

Supporting Information

Ultrasonic-Assisted Synthesis of N-Doped, Multicolor Carbon Dots toward Fluorescent Inks, Fluorescence Sensors, and Logic Gate Operations

Jiali Xu, Kai Cui, Tianyu Gong, Jinyang Zhang, Zhirou Zhai, Linrui Hou *, Fakhr uz Zaman and Changzhou Yuan *

School of Materials Science & Engineering, University of Jinan, Jinan 250022, China; Axujl_ME@163.com (J.X.); Acuik_ME@163.com (K.C.); Agongty_ME@163.com (T.G.); Azhangjy_ME@163.com (J.Z.); Azhaizr_ME@163.com (Z.Z.); Azaman_me@163.com (F.u.Z.)

* Correspondence: mse_houlr@ujn.edu.cn (L.H.) mse_yuancz@ujn.edu.cn (C.Y.)

Table S1. The atomic contents from the XPS data.

Samples	C	N	O
G-CDs	64.05%	2.74%	33.21%
C-CDs	79.21%	5.74%	15.05%
P-CDs	76.90%	1.72%	20.99%

Table S2. XPS data analysis of the C_{1s} spectra of three samples.

Samples	C = C/C-C	C-OH/C-N/C-O	C=O	COOH
G-CDs	44.22%	38.12%	9.88%	7.78%
C-CDs	78.60%	13.84%	3.04%	4.52%
P-CDs	67.95%	23.78%	3.92%	4.35%

Table S3. Fitting parameters and average PL lifetimes (τ) of the G-CDs, C-CDs, and P-CDs.

Sample	λ_{ex} (nm)	λ_{em} (nm)	lifetime						
			A_1	τ_1 (ns)	A_2	τ_2 (ns)	A_3	τ_3 (ns)	τ (avg)
G-CDs	360	473	1208.95	0.21	1355.77	1.62	2539.64	4.67	4.12
C-CDs	460	547	1256.57	0.33	2852.35	1.65	909.67	5.99	3.84
P-CDs	665	673	5089.96	6.18	-	-	-	-	6.18
